# Supplementary material for: Compensation of hyperexcitability with simulation-based inference
Source: eLife. 2026 Oct 30;14:RP107045. doi: 10.7554/eLife.107045 (PMC13722412; doi:10.7554/eLife.107045)
Supplement: Peer review history

# Editor report: eLife Assessment

**DOI:** 10.7554/eLife.107045.4.sa0

**Authors:** Inna Slutsky

**Affiliations:** Tel Aviv University Israel

**Keywords:** Convincing, Valuable

This study introduces a **valuable** simulation-based inference (SBI) framework to identify degenerate compensatory mechanisms that stabilize network activity despite neuronal hyperexcitability, a feature common to many brain disorders. By estimating posterior distributions of network parameters, the authors highlight factors such as threshold potential and interneuron-to-principal cell connectivity as key compensators for increased intrinsic excitability and interneuron loss. The approach is promising and can become a **convincing** tool for probing network degeneracy.

# Referee report: Joint Public Review

**DOI:** 10.7554/eLife.107045.4.sa1

[Editors' note: this version has been assessed by the Reviewing Editor without further input from the original reviewers. The authors have addressed the comments raised in the previous round of review.]

Summary:

This manuscript couples a 32-parameter model with simulation-based inference (SBI) to identify parameter changes that can compensate for three canonical hyperexcitability perturbations (interneuron loss, recurrent-excitatory sprouting, and intrinsic depolarisation). The study demonstrates a careful implementation of SBI and offers a practical ranking of "compensatory levers" that could, in principle, guide therapeutic strategies for epilepsy and related network disorders.

Strengths:

(1) By analysing three mechanistically distinct hyper-excitable regimes within the same modelling and inference framework, the work reveals how different perturbations require different compensatory interventions.

(2) The authors adopt posterior estimation to systematically rank the efficiency of different mechanisms in balancing hyperexcitability.

(3) Code and data are available.

# Author comment: Author response

**DOI:** 10.7554/eLife.107045.4.sa2

**Authors:** Daniel Müller-Komorowska, Tomoki Fukai

**Affiliations:** Okinawa Institute of Science and Technology Graduate University Tancha Japan; Okinawa Institute of Science and Technology Onna-son, Kunigami-gun Japan

The following is the authors’ response to the previous reviews.

**Joint Public Reviews:**

(1) The manuscript states that simulation-based calibration showed the amortized posterior estimator was unreliable (85-88), but these results are not shown. The manuscript explicitly states that simulation-based calibration demonstrated substantial failures of the amortized posterior estimator, yet the corresponding analyses are not presented. Since these results motivate the transition to sequential NPE and are central to assessing inference reliability, they should be reported quantitatively, either in the main text or supplementary material.

We agree and have therefore added the simulation-based calibration result as a supplementary figure.

(2) The authors present two independently trained estimators and show strong agreement between them. This is a useful robustness analysis. However, the rebuttal occasionally presents this as addressing concerns regarding cross-validation and generalization. The new analysis does not constitute cross-validation in the usual sense and does not directly assess generalization to held-out targets or posterior accuracy.

I recommend that the authors explicitly describe Figure 4 as a reproducibility analysis and avoid presenting it as a substitute for validation.

We are sorry for causing confusion with our response in the rebuttal. But we hope that the manuscript text is clearer than our rebuttal. We refer to the results as a “replication of the posterior estimator”. Regarding cross-validation, Figure 1 D shows additional simulations of posterior parameters that were not part of the training dataset.

(3) Posterior correlations are useful for generating hypotheses about compensatory mechanisms, but they should not be interpreted as direct evidence of compensation. The compensatory interpretation should instead be supported by the perturbation analyses (e.g., Figure 6), which provide mechanistic validation.

The manuscript consistently treats posterior correlations and conditional posterior shifts as direct evidence of compensatory mechanisms. These are consistent with compensatory mechanisms, but they do not by themselves establish that the corresponding biological parameters causally compensate for the perturbation. I recommend clarifying this distinction and emphasizing that the conditional posterior analyses generate hypotheses regarding compensation, which are then partially supported by the perturbation experiments shown later in the manuscript.

The language throughout the manuscript should therefore be softened.

We agree and have added hedging phrases to communicate the caveats of the results. For example, we now refer to “putative compensatory mechanisms” and adjusted the language in many other parts of the manuscript to highlight that our results provide estimates of mechanisms rather than the mechanisms themselves.

(4) The manuscript repeatedly suggests that the inferred conditional distributions may be useful for identifying precise interventions or guiding personalized treatments (examples include lines 24-29, lines 217-223, lines 242-246, lines 277-282, lines 283-286). These claims go beyond what is directly demonstrated.

The study does not evaluate treatment outcomes, patient-specific inference, intervention efficacy, or clinical decision-making. Rather, it demonstrates differences in inferred parameter distributions within a computational model. While these results are valuable and may generate clinically relevant hypotheses, they do not yet establish predictive utility for treatment selection or precision medicine. I therefore recommend substantially softening these translational claims and emphasizing that the current findings generate hypotheses that could be tested experimentally in future work.

You are correct. We are currently working to use our approach on patient data, but the present manuscript does not test the translational potential of the method and therefore some of our previous claims are unsupported. We have therefore deleted references to clinical potential in large parts of the manuscript and add qualifying phrases where we kept discussions of translational potential.

(5) The revised manuscript still mixes presentation of findings with interpretation.

For example, lines 217-226 largely continue to describe findings from Figure 6 and would fit better in the Results section. The Discussion would be strengthened by focusing more exclusively on biological implications, limitations, and future directions.

A similar issue appears later in the discussion comparing posterior correlations and conditional distributions. Much of this section effectively reinterprets Figures 2 and 3 rather than discussing broader implications.

We agree that the main function of the discussion is to put results into a broader context. But we also believe that repeating results at different levels of detail, different wording, and different interpretation can be to the benefit of some readers. We have therefore left the discussion largely intact except for the changes relating to above points 3 & 4.

(6) The discussion around lines 271-282 overstates what can be concluded from the inferred posteriors.

The statement that correlations "discover broadly applicable mechanisms" whereas conditionals "identify specific mechanisms" is stronger than the presented evidence supports. Likewise, the conclusion that conditional distributions are more useful for precision treatments is speculative and not directly demonstrated.

I recommend reformulating these statements as interpretations or hypotheses rather than conclusions.

We have deleted the reference to personalized treatment and refer to the mechanisms as “hypotheses” in other parts of the discussion.

(7) Around line 84, the manuscript introduces q(theta|x) without clearly defining θ, x, or q. Readers unfamiliar with SBI may struggle to follow the notation. All quantities should be defined when first introduced.

We have added definitions of theta and x. We hope that q is sufficiently defined when we call it the “posterior estimate”.

(8) The manuscript equates larger KS distances between conditional posteriors with greater compensatory potential. While KS distance provides a useful measure of posterior redistribution, it is not obvious that it should be interpreted as a measure of biological efficacy.

We hope that the changes described above make it clear that we view the KS distance as an estimate of putative compensatory mechanisms in the simulator, rather than direct measures of biological efficacy.

(9) The manuscript would benefit from a discussion of parameter identifiability. The inference problem maps 32 model parameters to 7 summary statistics, implying substantial nonidentifiability. While complete identifiability analysis is likely beyond the scope of the current work, this limitation should be discussed explicitly.

Non-identifiability is indeed an important aspect of simulation-based inference. We allude to it in the introduction where we mention degeneracy. But since we do not present results directly quantifying non-identifiability, we believe an explicit discussion goes beyond the scope of our manuscript.

All in all, the revised manuscript is significantly improved and addresses several concerns raised in the previous review. However, important issues remain as discussed above.

We thank you for the comments that led to the improved revision and for your constructive feedback on this revised version.
